# Integrative Analysis of Abiotic Stress–Responsive Genes in Soybean Using Differential Gene Expression and Validation With Machine Learning

**DOI:** 10.1002/pei3.70174

**Published:** 2026-06-22

**Authors:** Zohreh Hajibarat, Abbas Saidi

**Affiliations:** ^1^ Department of Cell and Molecular Biology, Faculty of Life Sciences and Biotechnology Shahid Beheshti University Tehran Iran

**Keywords:** abiotic stresses, common DEGs, DGCA, machine learning, soybean, XGBoost

## Abstract

The increasing frequency and intensity of climate‐associated abiotic stresses highlight the need to better understand soybean stress‐response mechanisms. In this study, transcriptomic data from 112 soybean samples exposed to multiple abiotic stress conditions, including drought, salinity, and heavy‐metal stress, were integrated using a multi‐layer computational framework. By combining differential expression analysis, Differential Gene Correlation Analysis (DGCA), Random Forest, XGBoost, functional enrichment, and network‐based interpretation, the analysis prioritized 37 candidate genes associated with abiotic stress responses across heterogeneous experimental contexts. Functional enrichment analyses indicated that these candidates were associated with amino acid biosynthesis, energy metabolism, carbon metabolism, glycolysis, secondary metabolite biosynthesis, and ROS‐related processes. DGCA further suggested stress‐associated changes in gene–gene correlation patterns, supporting the presence of condition‐dependent transcript coordination rather than direct causal regulation. Among the prioritized candidates, GLYMA_16G207700, GLYMA_16G204600, and GLYMA_16G214500 emerged as high‐priority genes based on multi‐layer analytical support, chromosomal localization, and comparative genomic evidence. Their orthology or syntenic relationships with loci in *Glycine soja, Phaseolus vulgaris,* and *Vigna angularis* suggest that some components of the soybean abiotic stress‐response signature may be conserved across related legumes. Overall, these findings provide computationally derived candidate genes and testable hypotheses for future functional validation, haplotype–phenotype association analysis, and data‐informed breeding studies aimed at improving soybean stress resilience.

## 1 Introduction

Soybean, scientifically classified as *Glycine max* (L.) Merr., serves as a cornerstone legume crop globally, providing essential vegetable oil and protein for both human nutrition and animal feed (Kumari et al. 2023). However, its economic significance is tempered by persistent challenges from environmental stressors. These include drought, salt fluctuations, temperature extremes, heavy metal exposure, and soil nutrient imbalances (Dong et al. 2019; Oyebamiji et al. 2024). Notably, drought has historically impacted approximately 67 million hectares of soybean cultivation between 1983 and 2009, causing an estimated 7% reduction in yield (Song et al. 2022). Collectively, abiotic stress is thought to contribute to over half of crop losses worldwide, posing a critical risk to feeding global populations and sustaining livestock (Lesk et al. 2020).

When plants confront environmental hardships, they undergo profound metabolic and genetic adjustments. Central to this adaptation is carbohydrate metabolism, which orchestrates carbon allocation, energy generation, and the synthesis of secondary metabolites crucial for defense mechanisms. Research has shown that sugars function not only as metabolic substrates but also as regulatory signals influencing stress‐related gene activity, as observed in rice and other food crops (Kumari et al. 2022; Mathan et al. 2021). These findings underscore the interconnectedness of core metabolic processes and gene regulation in stress resilience.

High‐throughput RNA sequencing (RNA‐seq) has revolutionized the study of transcriptional responses to environmental stress. Yet, analyzing these datasets remains complex due to their vast size, technical inconsistencies, and limited biological replicates relative to the number of genes analyzed (Chandrashekar et al. 2024; Farooq et al. 2024). These constraints hinder the reliable identification of key stress‐responsive genes across independent research. To overcome this, integrative strategies like meta‐analysis are increasingly utilized to enhance statistical robustness and uncover shared transcriptional patterns across diverse datasets and experimental conditions (Yu et al. 2025; Rosenthal and Rubin 1986; Sanches et al. 2024).

Recent advancements in artificial intelligence, particularly machine learning (ML), have offered novel solutions for transcriptomic analysis. These techniques excel in pinpointing critical regulatory genes linked to traits like stress tolerance by evaluating predictive relevance beyond mere expression levels. Tools such as Random Forest (RF) and XGBoost allow for prioritizing genes based on their functional impact. Simultaneously, network‐based methods like Differential Gene Correlation Analysis (DGCA) complement these efforts by mapping changes in gene regulatory interactions that expression data alone might miss (Ghazal et al. 2024; McKenzie et al. 2016).

Despite these innovations, most studies to date have applied these computational methods in isolation, leaving gaps in understanding the intricate regulatory networks governing stress responses. Specifically, integrative frameworks that merge ML‐driven feature selection with differential correlation and cross‐species evolutionary analyses are underrepresented in soybean research.

To address this, our study developed a combined analytical strategy integrating Random Forest, XGBoost, and DGCA to uncover consistent abiotic stress–responsive genes in soybean using publicly available RNA‐seq datasets. Additional analyses, including functional enrichment, protein interaction networks, chromosomal location studies, and syntenic comparisons across species, were conducted to define the roles and evolutionary conservation of these genes. By synthesizing complementary computational methodologies, this work illuminates the genetic and regulatory underpinnings of soybean's response to environmental stressors and highlights potential targets for improving crop resilience.

## 2 Material and Methods

### 2.1 Compilation of RNA‐Seq Resources

Publicly accessible soybean (Glycine max) RNA‐seq experiments that explore reactions to abiotic stresses were downloaded from the NCBI Sequence Read Archive (SRA) (see Table 1). Ten separate RNA‐seq projects, encompassing 112 individual libraries, were chosen. These datasets span a wide spectrum of stress treatments such as drought, polyethylene glycol (PEG), a PEG and salt regimen, phosphorus shortage, salt, cadmium exposure, nitrogen deficiency, aluminum toxicity, and sugar application. By integrating several independent investigations, we could capture both universally conserved and stress‐specific transcriptional patterns across varied environmental scenarios.

**TABLE 1:** Characteristics of RNA‐seq datasets selected for meta‐analysis, including accession number, stress type, treated sample conditions, tissue type, total number of samples, and reference.

Tissues	Number of samples	Type of stress	Accession	References	Group
Seedling	9	Drought	PRJNA813355	Xuan et al. (2022)	B
Leaves	12	PEG	PRJNA823397	Wang et al. (2022)	D
Leaves	11	PEG	PRJNA957077	Li et al. (2024)	E
Leaves	15	PEG and Salt	PRJNA930177	Du et al. (2023)	I
Leaves	6	Aluminum toxicity	PRJNA591095	Zhao et al. (2020)	H
Leaf	18	Salt	PRJNA1110806	Fu et al. (2024)	G
Root	8	Cadmium stress	PRJNA728527	Ma et al. (2022)	A
Root	6	Nitrogen deficiency	PRJNA1244565	Chen et al. (2025)	C
Root	9	Sugar treatment	PRJNA951790	—	J
Root	18	Phosphorus deficiency	PRJNA867332	Yang et al. (2022)	F

### 2.2 Quality Assessment and Data Cleaning

The raw Illumina reads underwent an initial quality check with FastQC (v0.11.8). Subsequent trimming of adapters, ambiguous bases, and low‐quality nucleotides (Phred < 20) was performed using Trimmomatic (v0.32). A second FastQC run confirmed that only high‐quality reads proceeded to the downstream workflow.

### 2.3 Alignment and Count Generation

Cleaned reads were mapped to the soybean reference genome (*Glycine max* v2.1) employing HISAT2, a splice‐aware aligner suited for RNA‐seq data. Resulting SAM/BAM files were sorted and indexed with SAMtools. Gene‐level read counts were extracted via featureCounts, restricting the analysis to uniquely aligned reads to obtain reliable expression estimates for every sample.

### 2.4 Detection of Differentially Expressed Genes

The raw count matrix was normalized and interrogated with DESeq2, which models read counts through a negative‐binomial distribution to accommodate both biological and technical variability. Genes showing significant changes for each stress condition were identified using a false‐discovery‐rate (FDR) cutoff of 0.05. DEGs were defined using FDR < 0.05 and |log2FC| > 1. Each stress condition was analyzed independently before meta‐analysis.

### 2.5 Meta‐Analysis and Correction of Batch Effects

To pinpoint genes consistently regulated across the different studies, a meta‐analysis was carried out with the metaRNASeq package, merging *p*‐values from the individual DESeq2 results. Batch effects stemming from disparate experimental designs and sequencing platforms were mitigated with the ComBat function from the sva package. This correction was applied to variance‐stabilized expression data before integration, preserving genuine biological signals while suppressing technical noise. Before proceeding with further analysis, we assessed and mitigated batch effects within the dataset. We began by performing Principal Component Analysis (PCA) and Uniform Manifold Approximation and Projection (UMAP) on the normalized data to examine both local and global sample structures and to detect any unintended technical discrepancies. By overlaying sample metadata, we scrutinized the clusters to determine if they aligned more strongly with batch origins than with the expected biological variables. To address these artifacts, we employed a linear modeling approach that accounted for batch as a technical covariate. Throughout this adjustment, we incorporated biological group information into the model to ensure that meaningful biological signals remained intact. Once the correction was applied, we re‐ran the PCA and UMAP diagnostic plots. We deemed the normalization successful if the samples showed increased inter‐batch mixing and a reduction in batch‐driven clustering, all while preserving the integrity of the biological groupings. The efficacy of this correction was validated by visually comparing the pre‐ and post‐adjustment PCA and UMAP plots. While PCA served to illustrate the primary drivers of variance, UMAP provided a detailed view of the non‐linear associations between samples.

### 2.6 Further Integrative and Functional Analyses

The set of common DEGs uncovered by the meta‐analysis was subjected to functional enrichment examinations using Gene Ontology (GO) terms and KEGG pathways. Differential Gene Correlation Analysis (DGCA) was used to explore how stress conditions reshape gene–gene correlation networks. Concurrently, we utilized machine learning‐based feature selection to identify candidate genes associated with abiotic stress responses, thereby creating a computational prioritization framework for understanding the transcriptional mechanisms governing soybean responses to environmental challenges.

### 2.7 Stress‐Induced Transcriptional Pattern Recognition via Machine Learning

To identify and anticipate gene expression shifts under abiotic stress conditions, two supervised learning algorithms were constructed and analyzed using R: Random Forest (RF) and eXtreme Gradient Boosting (XGBoost).

### 2.8 Preprocessing and Dimensionality Reduction

To refine the dataset and enhance computational efficiency, genes exhibiting variance below the 90th percentile threshold were excluded. This process retained 1916 genomic features across 112 biological replicates after standardization. A five‐fold cross‐validation with explicit hyperparameter tuning grids was adopted to fine‐tune parameter configurations and assess model adaptability to new data.

### 2.9 Labeling Strategy

Samples were annotated using a binary framework:

- Stress‐exposed samples → classification label: 0
- Control (unstressed) samples → classification label: 1This binary labeling system formed the basis for training the predictive models to distinguish stress‐associated patterns.

### 2.10 Algorithm Implementation

#### 2.10.1 Random Forest (RF)

The RF ensemble approach combined 500 decision trees with Gini index splitting criteria to mitigate overfitting risks. To balance class distribution, inverse‐frequency weighting was applied, and tree depths were capped at five nodes. This configuration balanced model simplicity with robustness, preserving interpretability through gene‐ranking importance metrics.

#### 2.10.2 eXtreme Gradient Boosting (XGBoost)

XGBoost enhanced predictive accuracy through iterative tree construction with regularization penalties. Key optimized parameters included:

- Nrounds (iteration count for boosting).
- Eta (adjustment rate for step size).
- Subsample (fraction of data used per boosting cycle).
- Colsample_bytree (feature sampling ratio per decision tree). Gene ranking was derived from XGBoost's intrinsic importance metrics, highlighting biologically significant markers.

Principal Component Analysis (PCA) was applied to the gene‐expression matrix to reveal latent structure and highlight coordinated transcriptional programs underlying the stress response. Model performance was evaluated using overall accuracy and AUC, ensuring that both class‐level correctness and ranking ability were captured. By integrating multiple learning algorithms, this ensemble‐style strategy exploited complementary inductive biases to identify stress‐associated transcriptional patterns while preserving biological interpretability through feature‐level insights.

### 2.11 Unraveling Gene Interactions Under Abiotic Stress

To decipher the complex relationships between genes, we employed a differential gene correlation analysis (DGCA) approach. By utilizing the DGCA package in R, we examined the correlations between gene pairs under control and abiotic stress conditions (McKenzie et al. 2016). The correlation coefficients were then transformed into z‐scores using the Fisher z‐transformation, which involves a mathematical operation that stabilizes the variance of the correlations. This transformation is represented by the equation:

$$z=a\tanh\left(r\right)=\frac{1}{2}\log_{e}\left(\frac{1+r}{1-r}\right)$$

where *r* is the correlation coefficient, and *a*tanh denotes the inverse hyperbolic tangent function (Fieller et al. 1957). The variance of the resulting *z*‐scores depends on the type of correlation (Pearson or Spearman) and can be calculated using the formula:

$$\mathrm{var}\left(r_{p}\right)=\frac{1}{n-3}\;\text{or}\;\mathrm{var}\left(r_{s}\right)=\frac{1.06}{n-3}$$

where *n* is the sample size. We then calculated the difference in *z*‐scores (*d*_*z*_) between control and stress conditions, which is given by:

$$d_{z}=\frac{\left(z_{1}-z_{2}\right)}{\sqrt{\left|S_{z_{1}}^{2}-S_{z_{2}}^{2}\right|}}$$

Using this *d*_*z*_. value, we computed a two‐sided *p*‐value based on the standard normal distribution and ranked the gene pairs according to their differential correlation values.

### 2.12 Functional Enrichment and Network Analysis

To visualize the overlap between differentially expressed genes (DEGs) identified by machine learning models, we generated Venn diagrams using the VennDiagram package in R. We also performed Gene Ontology (GO) enrichment analysis on the shared genes among the three models, focusing on biological processes, cellular components, and molecular functions using the ShinyGO database.

Furthermore, we analyzed the distribution of shared genes across different chromosomes using density plots and investigated protein–protein interactions among DEGs using the STRING database. We filtered out interactions with confidence scores below 0.4 to ensure reliability.

### 2.13 Validation and Predictive Analysis

To validate our findings, we conducted synteny analysis across four legume species—*Glycine max*, *Phaseolus vulgaris* , *Vigna angularis*, and *Glycine soja—to* identify orthologous gene clusters and conserved chromosomal segments associated with stress adaptation. This approach leverages genomic context to improve classification accuracy for ortholog detection.

We also evaluated the predictive potential of three genes—GLYMA_16G207700, GLYMA_16G204600, and GLYMA_16G214500—using receiver operating characteristic (ROC) analysis. ROC curves were visualized using ggplot2, and the area under the curve (AUC) was calculated using the pROC package. AUC scores above 0.7 were considered indicative of notable predictive power.

## 3 Results

**FIGURE 1:**
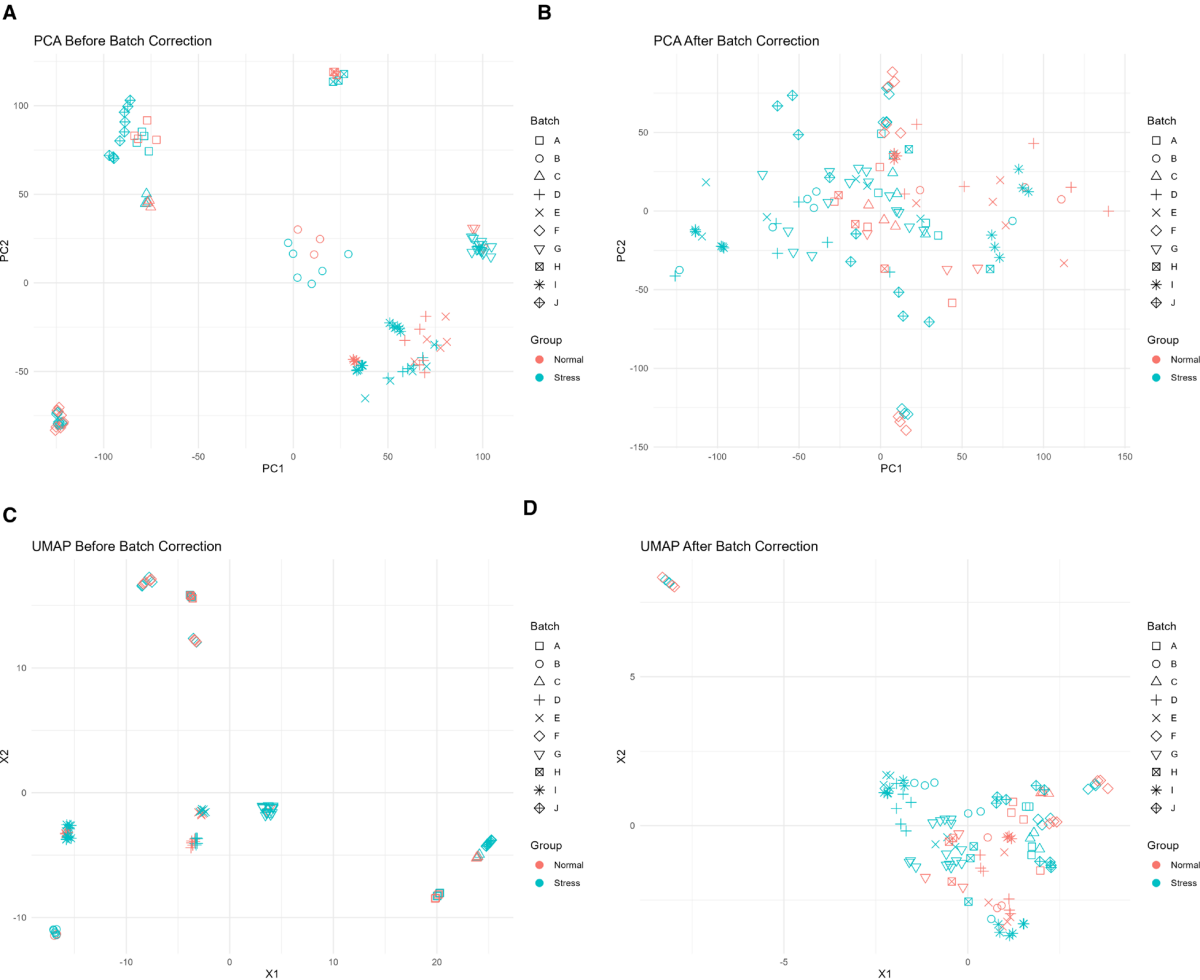
Visualization of batch effects (BE) and their correction. (A) PCA before batch correction; (B) PCA after batch correction; (C) UMAP before batch correction; (D) UMAP after batch correction.

Upon combining 112 samples from 10 diverse RNA‐Seq datasets, noticeable discrepancies emerged, as illustrated in Figure 1A,B. These discrepancies stem from technical, rather than biological, factors—including differences in sequencing technologies, library preparation methods, sample processing, and experimental environments.

A closer examination of the boxplot in Figure 1A reveals that the median values of the distributions are scattered, pointing to the presence of technical variability between samples. However, after applying batch correction, a significant improvement is observable: the median values in Figure 1B show greater consistency across samples, indicating that the technical variability has been substantially reduced. This visualization underscores the critical need to account for technical differences when analyzing multiple datasets, ensuring that the results reflect genuine biological patterns rather than artificial variations.

### 3.1 Venn‐Diagram Overview

Expression profiles of soybean subjected to abiotic stress were compiled, revealing 1916 differentially expressed genes (DEGs) through a meta‐analytic approach. The relative importance of these genes was assessed with four algorithms: Random Forest (RF), XGBoost models, and the DGCA framework.

### 3.2 Consensus Gene Set

**FIGURE 2:**
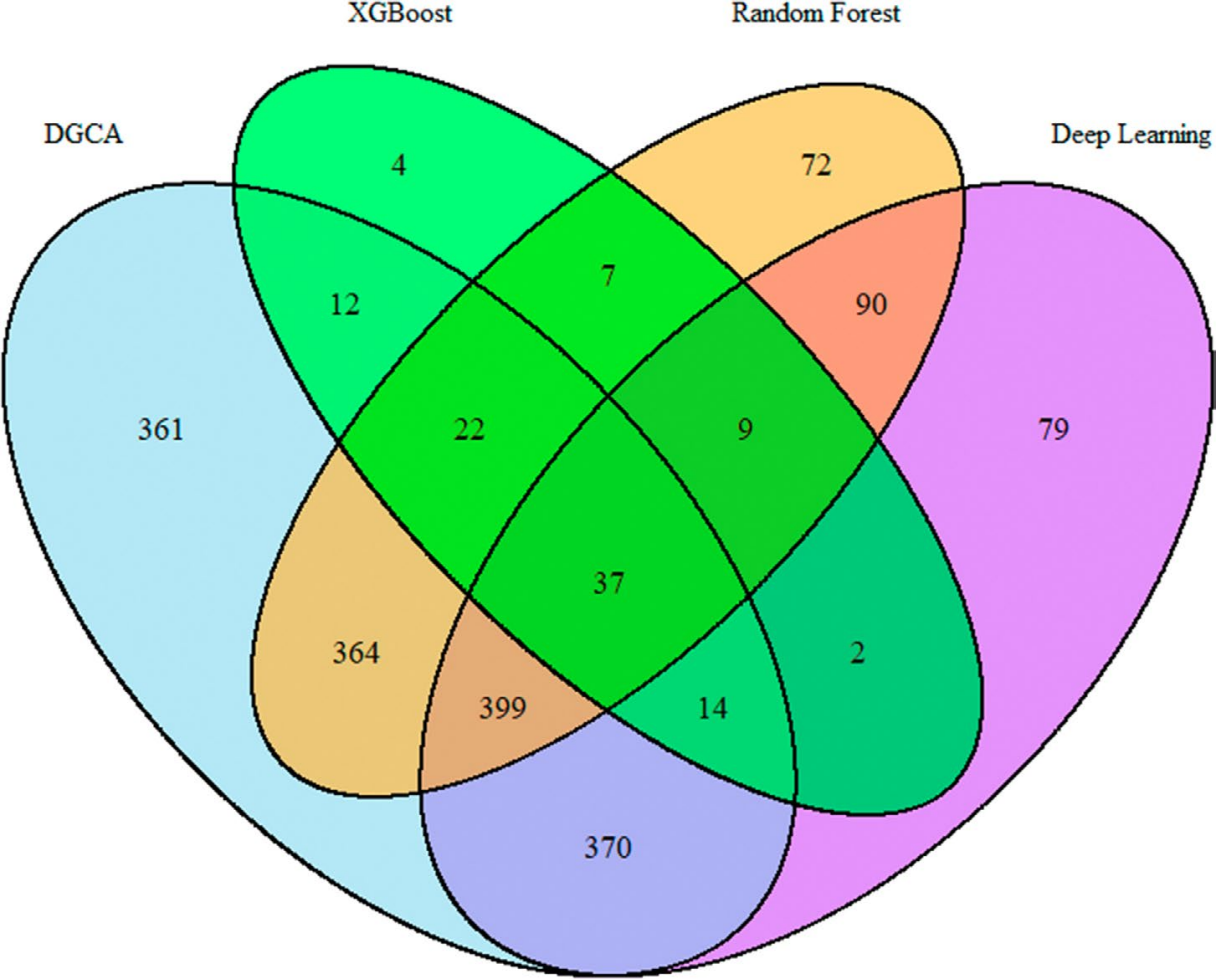
Venn diagram analysis of common DEGs across two machine learning algorithms (RF and XGBoost) and DGCA.

Applying a gain‐oriented feature selection filter across all four methods yielded a core set of 37 genes that consistently contributed to classification accuracy (see Figure 2). Because these loci repeatedly emerged as top predictors, they are regarded as candidate genes associated with the plant's response to salt stress.

### 3.3 Model Validation Strategy

To guarantee the robustness of the predictive models, a 5‐fold cross‐validation routine was executed and the whole process was repeated 100 times. This extensive resampling reduces the influence of any particular data split, delivers a reliable estimate of model performance, and strengthens confidence in the transferability of the selected features. The repeated validation confirmed that the models retained high predictive accuracy with minimal variance, underscoring the utility of the 37 common genes as reliable molecular markers for stress classification and genotype evaluation.

### 3.4 List of the 37 Shared Genes

GLYMA_19G227800, GLYMA_08G211900, GLYMA_20G124200, GLYMA_02G228300, GLYMA_16G207700, GLYMA_08G328400, GLYMA_11G170300, GLYMA_16G204600, GLYMA_01G190300, GLYMA_04G021000, GLYMA_18G030700, GLYMA_07G229100, GLYMA_18G009700, GLYMA_05G070400, GLYMA_08G154300, GLYMA_03G132700, GLYMA_17G127900, GLYMA_03G098600, GLYMA_05G108200, GLYMA_02G018700, GLYMA_07G155500, GLYMA_06G021200, GLYMA_09G185500, GLYMA_01G127200, GLYMA_15G021400, GLYMA_11G121800, GLYMA_08G318400, GLYMA_14G223000, GLYMA_03G173300, GLYMA_16G123400, GLYMA_16G214500, GLYMA_10G267700, GLYMA_05G169900, GLYMA_08G199300, GLYMA_06G137000, GLYMA_09G049100, GLYMA_06G315100.

### 3.5 Chromosomal Insight

Notably, three of these genes are positioned on soybean chromosome 16, suggesting that this region may serve as a focal point for loci that mediate stress‐responsive mechanisms.

### 3.6 Genomic Localization of Shared Differentially Expressed Genes

**FIGURE 3:**
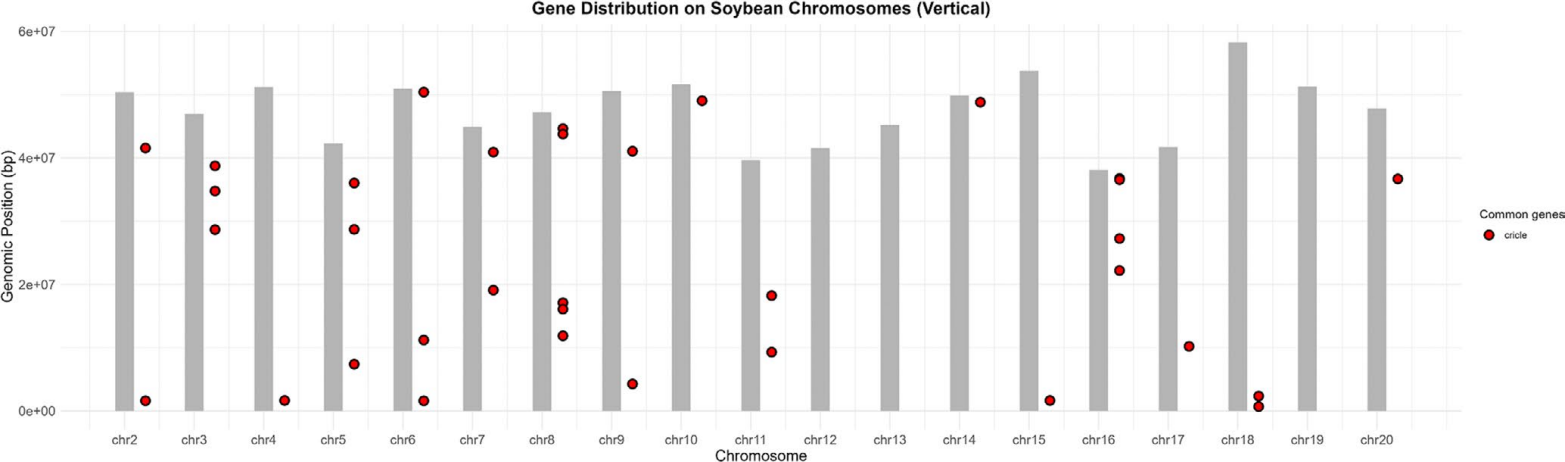
Distribution of novel and 37 common DEGs in 20 soybean chromosomes.

Visualized in Figure 3, the chromosomal distribution of 37 commonly identified differentially expressed genes (DEGs) was mapped using the ggplot2 visualization toolkit. A majority of these genes were localized to specific chromosomes, with comprehensive distribution details visualized in Figure 2. A detailed examination of gene positions revealed that chromosomes 8 and 16 contained the highest concentration of genes linked to salt stress responses. This overrepresentation implies the presence of enriched genomic regions or areas with heightened transcriptional activation under abiotic stress conditions. Conversely, other chromosomes exhibited a more even distribution of genes, suggesting widespread genetic participation without distinct clustering. The observed gene distribution may be attributed to variations in chromosomal structure, differences in gene density, or the presence of regulatory sequences specialized for stress adaptation. Fisher's exact tests confirmed a significant enrichment of candidate genes on chromosomes 8 and 16 (chr8: *p* = 6.58 × 10^−10^; chr16: *p* = 1.59 × 10^−11^), indicating that the observed clustering on these chromosomes is unlikely to be random and is consistent with localized genomic organization of functionally related genes. Computational pipeline for integrative identification of soybean abiotic stress candidate genes. The following R script summarizes the main computational workflow used to prioritize candidate genes associated with abiotic stress responses in soybean. The workflow integrates differential expression analysis, differential gene correlation analysis, and two supervised machine learning algorithms, Random Forest and XGBoost. The final candidate genes were defined as the intersection of genes identified across DGCA, Random Forest, and XGBoost analyses (Data S1).

### 3.7 GO and KEGG Functional Investigation

**FIGURE 4:**
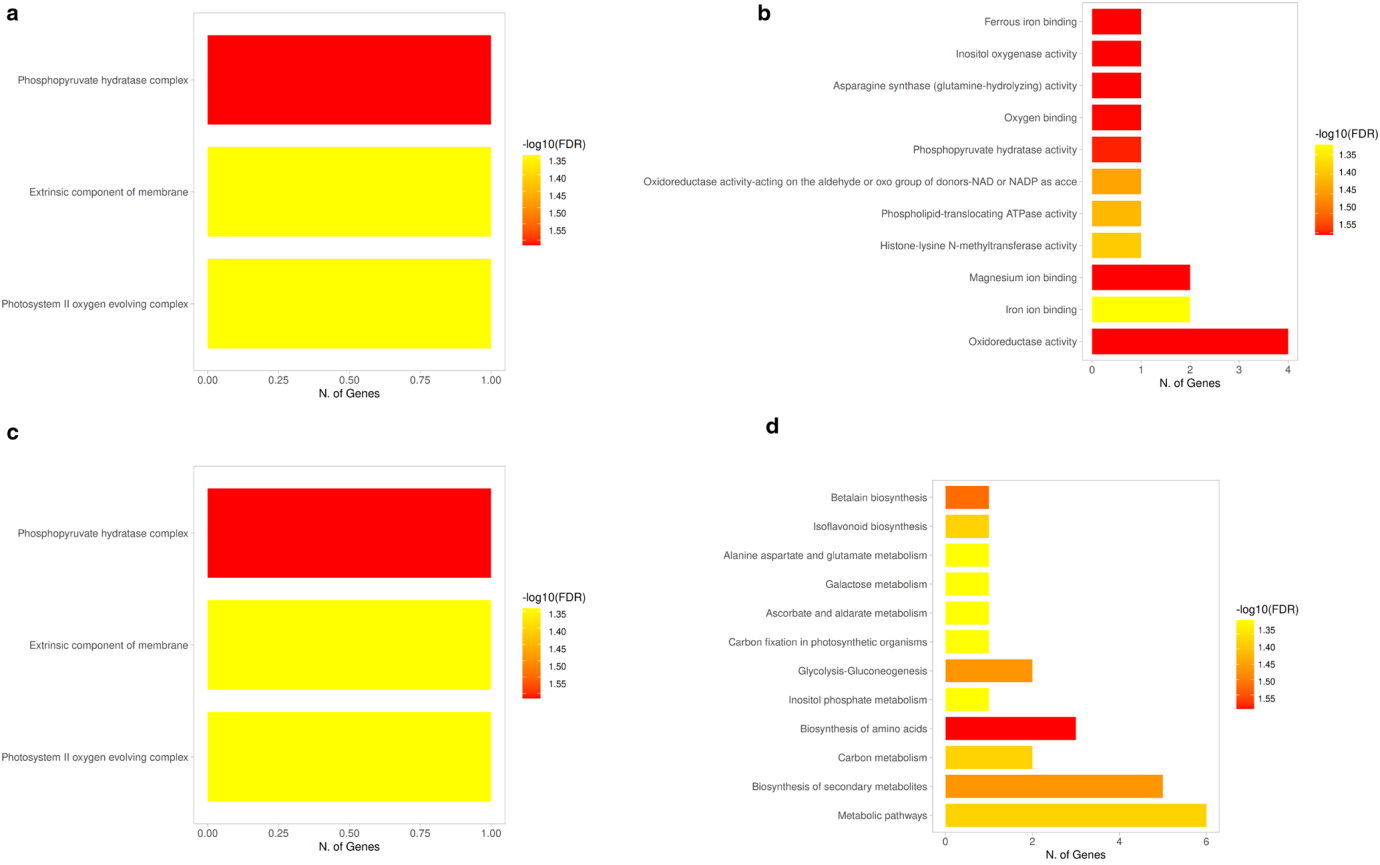
Predicted functions of common DEGs based on GO and KEGG terms in the soybean genome. The *x*‐axis represents the number of common genes associated with each function shown on the *y*‐axis. The size of the points in the bar plot indicates the corresponding FDR. (a) Biological‐process, (b) molecular‐function, (c) cellular components, (d) KEGG analysis of common genes.

To uncover the biological significance of the 37 overlapping differentially expressed genes (DEGs) detected in the various data sets, we carried out Gene Ontology (GO) enrichment testing. The analysis covered the three classic GO domains: biological processes (BP), molecular functions (MF), and cellular components (CC). As shown in Figure 4, the most strongly enriched GO terms demonstrate that these genes largely cluster within essential pathways, activities, and cellular structures that mediate the plant's reaction to salt stress.

#### 3.7.1 Biological‐Process Insights

The GO‐BP results highlighted a suite of metabolic and energy‐related routes, such as glycolysis, oxidoreductase reactions, and the synthesis of amino acids. Notably, the enzyme enolase emerged as a pivotal node linking amino acid biosynthesis, the glycolysis‐gluconeogenesis axis, and the generation of secondary metabolites (Figure 4a).

#### 3.7.2 Molecular‐Function Profile

A number of MF terms signaled a tight connection with mechanisms of abiotic stress tolerance, including:

- GO:0008198—Ferrous‐iron binding.
- GO:0016491—General oxidoreductase activity.
- GO:0050113—Inositol‐oxygenase activity.
- GO:0000287—Magnesium‐ion binding.
- GO:0004066—Asparagine‐synthase (glutamine‐hydrolyzing) activity.
- GO:0019825—Oxygen binding.
- GO:0004634—Phosphopyruvate‐hydratase activity.
- GO:0016620—Oxidoreductase activity (aldehyde/oxo‐group donors with NAD/NADP as acceptors).
- GO:0004012—Phospholipid‐translocating ATPase activity.

Together, these functions point to a layered defensive scheme that integrates ion homeostasis, metabolic re‐programming, and reactive‐oxygen‐species (ROS) scavenging—critical components of abiotic stress resilience (Figure 4b).

#### 3.7.3 Additional Enriched Processes

Further GO‐BP terms associated with the common DEGs comprised:

- GO:0006529—Asparagine biosynthetic process
- GO:0006725—Metabolism of cellular aromatic compounds
- GO:0009415—Response to water
- GO:0019310—Inositol catabolic process
- GO:0055114—Oxidation–reduction process

#### 3.7.4 Cellular‐Component Distribution

The CC analysis indicated that several genes belong to the phosphopyruvate‐hydratase complex, suggesting a role in downstream signaling cascades initiated by chemical cues (Figure 4c). These compartments are linked to redox reactions, stress‐responsive pathways, and asparagine production.

#### 3.7.5 KEGG Pathway Mapping

KEGG enrichment revealed that soybean mobilizes a variety of essential pathways to mitigate abiotic stress, notably:

- Amino acid biosynthesis.
- Betalain biosynthesis.
- Glycolysis‐gluconeogenesis.
- Biosynthesis of secondary metabolites (See Figure 4d).

Both GO and KEGG examinations show that the 37 shared DEGs are embedded in coordinated metabolic and regulatory networks that underpin stress tolerance. Their consistent involvement across multiple functional categories underscores their potential as molecular markers for breeding programs aimed at enhancing stress‐resilient soybean varieties.

### 3.8 Dynamic Gene Network Analysis Through Correlation Shifts (DGCA)

To develop a biologically relevant predictive framework, it is crucial to not only assess individual gene expression levels but also investigate interactions between genes. A powerful strategy for this involves dynamic gene correlation analysis (DGCA), which scrutinizes variations in the relationship strength between gene pairs across contrasting scenarios, such as unstressed versus abiotic stress conditions. This approach unveils regulatory reconfigurations, highlighting co‐regulation events, pathway adjustments, or shifts in transcriptional activity triggered by environmental challenges.

In this research, pairwise gene interactions were examined for 37 overlapping differentially expressed genes (DEGs) identified through methods including random forest (RF), XGBoost, and DGCA. The analysis revealed statistically significant correlation alterations: 18, 3, 7, and 9 gene pairs demonstrated *p*‐values under 0.05, 0.01, 0.005, and 0.001 thresholds, respectively (Table 2), signifying substantial regulatory reprogramming.

**TABLE 2:** A pairwise analysis was conducted on soybean samples under control and abiotic stress conditions using the DGCA package to examine the correlation differences between gene pairs.

Gene1	Gene2	Normal_cor	Normal_*p*Val	Stress_cor	Stress_*p*Val	*z*ScoreDiff	*p*ValDiff	*p*ValDiff_adj	Classes
GLYMA_19G227800	GLYMA_17G242900	0.9498	0	0.4096	0.0003	−6.801	1.04E−11	0.00000119	+/+
GLYMA_08G211900	GLYMA_13G154900	0.9589	0	0.6273	3E−09	−5.829	5.59E−09	0.0000892	+/+
GLYMA_20G124200	GLYMA_08G200100	−0.929	0	−0.459	4E−05	5.6119	2E−08	0.00017403	−/−
GLYMA_02G228300	GLYMA_20G022500	0.9477	0	0.5935	3E−08	−5.488	4.06E−08	0.00027786	+/+
GLYMA_16G207700	GLYMA_16G021000	0.8095	4E−10	0.0392	0.7417	−5.296	1.18E−07	0.00054439	+/0
GLYMA_08G328400	GLYMA_16G021000	−0.225	0.1677	0.6935	1E−11	5.2851	1.26E−07	0.00056211	0/+
GLYMA_11G170300	GLYMA_11G078300	0.9414	0	0.5939	3E−08	−5.198	2.01E−07	0.0007471	+/+
GLYMA_16G204600	GLYMA_16G021000	0.8739	4E−13	0.3203	0.0057	−4.961	7.01E−07	0.00154767	+/+
GLYMA_01G190300	GLYMA_09G094000	0.939	0	0.6262	3E−09	−4.849	1.24E−06	0.00216003	+/+
GLYMA_04G021000	GLYMA_16G096600	0.7844	3E−09	0.9665	0	4.7744	1.8E−06	0.00262562	+/+
GLYMA_18G030700	GLYMA_01G099800	0.2082	0.2033	−0.643	8E−10	−4.756	1.98E−06	0.00277676	0/−
GLYMA_07G229100	GLYMA_12G210600	−0.921	0	−0.552	4E−07	4.7451	2.08E−06	0.00288185	−/−
GLYMA_18G009700	GLYMA_09G094000	0.9124	7E−16	0.5338	1E−06	−4.615	3.93E−06	0.00410495	+/+
GLYMA_05G070400	GLYMA_16G021000	0.607	4E−05	−0.213	0.0698	−4.49	7.12E−06	0.00576705	+/0
GLYMA_08G154300	GLYMA_11G198500	−0.945	0	−0.699	6E−12	4.4769	7.57E−06	0.00597152	−/−
GLYMA_03G132700	GLYMA_11G042200	0.1744	0.2884	−0.623	4E−09	−4.42	9.86E−06	0.00698079	0/−
GLYMA_17G127900	GLYMA_17G084600	0.5788	0.0001	0.9143	0	4.3521	1.35E−05	0.00832324	+/+
GLYMA_03G098600	GLYMA_07G091600	0.883	1E−13	0.4642	4E−05	−4.322	1.54E−05	0.00897024	+/+
GLYMA_05G108200	GLYMA_12G094100	0.9271	0	0.6516	4E−10	−4.191	2.78E−05	0.01217788	+/+
GLYMA_02G018700	GLYMA_16G021000	0.7023	6E−07	0.9368	0	4.0926	4.26E−05	0.01542426	+/+
GLYMA_07G155500	GLYMA_07G141300	0.9409	0	0.7304	2E−13	−3.981	6.86E−05	0.01970409	+/+
GLYMA_06G021200	GLYMA_14G162100	0.7896	2E−09	0.9544	0	3.944	8.01E−05	0.02136023	+/+
GLYMA_09G185500	GLYMA_08G200100	−0.372	0.0197	0.3787	0.001	3.8488	0.000119	0.02612501	−/+
GLYMA_01G127200	GLYMA_15G199700	0.8853	7E−14	0.5565	3E−07	−3.764	0.000167	0.03145713	+/+
GLYMA_15G021400	GLYMA_16G021000	−0.396	0.0126	0.3318	0.0041	3.7236	0.000196	0.03421401	−/+
GLYMA_11G121800	GLYMA_11G078300	0.6118	4E−05	−0.034	0.7742	−3.637	0.000276	0.04099417	+/0
GLYMA_08G318400	GLYMA_16G021000	−0.099	0.5476	0.5675	2E−07	3.6249	0.000289	0.04206227	0/+
GLYMA_14G223000	GLYMA_08G200100	0.6111	4E−05	−0.025	0.8345	−3.586	0.000335	0.04544679	+/0
GLYMA_03G173300	GLYMA_11G144600	0.9684	0	0.8694	0	−3.583	0.000339	0.04574789	+/+
GLYMA_16G123400	GLYMA_16G021000	0.5308	0.0005	−0.135	0.2561	−3.543	0.000395	0.04975692	+/0
GLYMA_15G199700	GLYMA_16G214500	0.892743	2.24E−14	0.406469	0.000359	−4.89485	9.84E−07	0.001886001	+/+
GLYMA_18G231500	GLYMA_10G267700	0.965378	0	0.828106	0	−4.08303	4.45E−05	0.015715453	+/+
GLYMA_01G099800	GLYMA_05G169900	0.135626	0.410372	−0.58135	6.93E−08	−3.90533	9.41E−05	0.023261027	0/−
GLYMA_10G282200	GLYMA_08G199300	0.646061	8.91E−06	−0.01584	0.894186	−3.82434	0.000131123	0.027533248	+/0
GLYMA_10G145300	GLYMA_06G137000	0.260979	0.108552	0.77192	1.33E−15	3.695394	0.000219546	0.03634007	0/+
GLYMA_15G199700	GLYMA_09G049100	0.83339	4.58E−11	0.423908	0.000186	−3.6407	0.000271898	0.040670469	+/+
GLYMA_18G231500	GLYMA_06G315100	0.967321	0	0.865002	0	−3.58846	0.000332642	0.045240199	+/+

*Note:* In the analysis, a total of 37 genes were included, selected through a combination of four methods and common DEGs identification. A significance threshold with *p* value of 0.05 was applied, resulting in a total of pairwise comparisons. Among these comparisons, the top 37 gene pairs demonstrated a heightened level of significance, with a *p* value of 0.05.

Abbreviations: Normal_cor, Normal Correlation Coefficient; Normal_*p*Val, Normal *p*‐Value; *p*ValDiff, *p*‐Value of Difference; *p*ValDiff_adj, Adjusted *p*‐Value of Difference; Stress_cor, Stress Correlation Coefficient; Stress_*p*Val, Stress *p*‐Value; *z*ScoreDiff, *z*‐Score Difference.

#### 3.8.1 Categorization of Gene Pair Interactions

**FIGURE 5:**
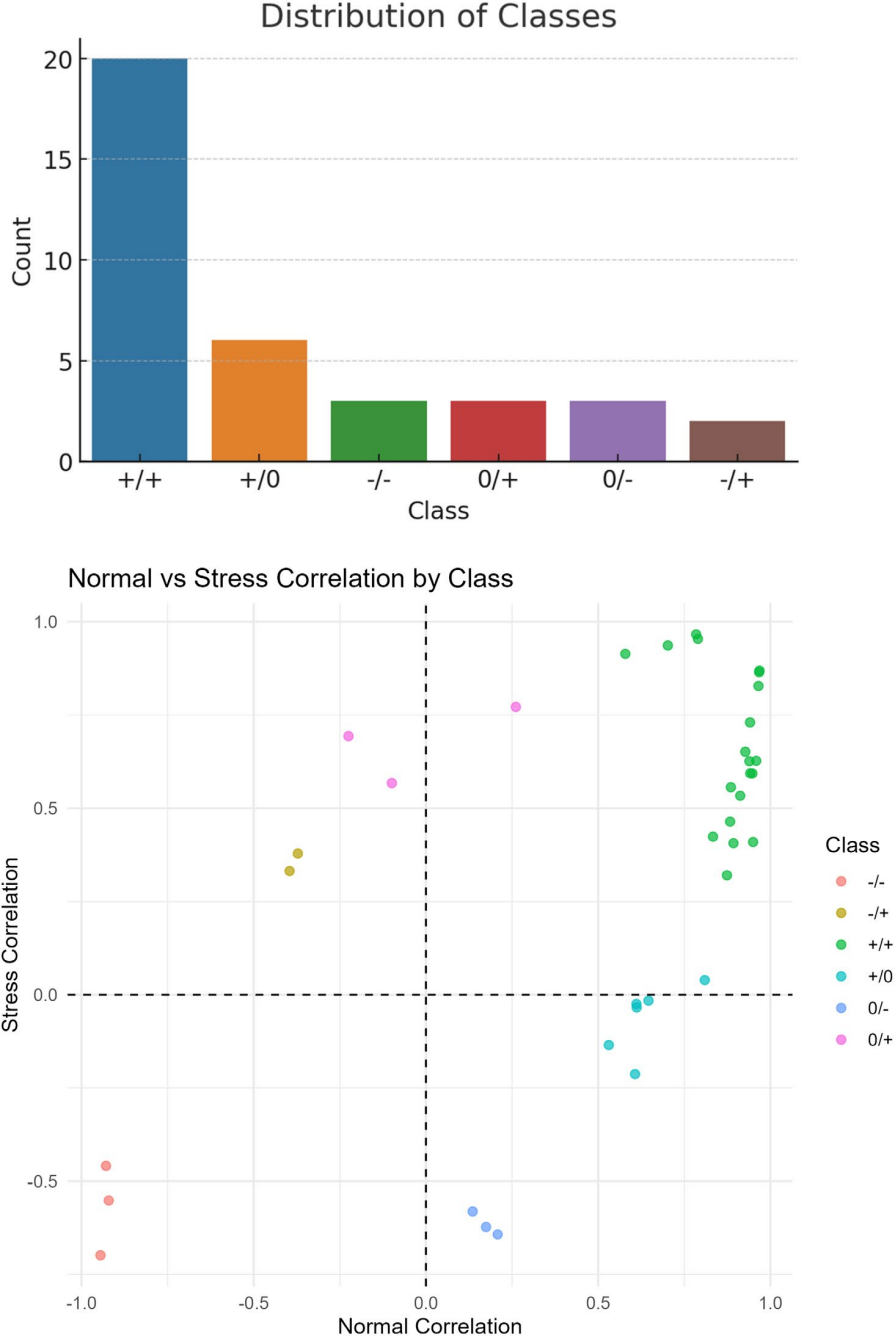
Differential correlation analysis of the 37 common soybean genes using DGCA.

The differentially correlated gene pairs were grouped into six classes based on their directional relationship shifts between conditions (Figure 5):

1. +/+: Maintained a positive association under both conditions.
2. +/−: Transitioned from a positive to a negative correlation.
−/−: Retained a negative correlation in both scenarios.
−/+: Shifted from a negative to a positive relationship.
5. 0/+: Developed a positive correlation exclusively under stress.
6. 0/−: Formed a negative correlation specifically under stress.

For instance, six gene pairs exhibited positive interactions in standard conditions that faltered under stress (+/0), while three pairs lacked correlation under normal conditions but aligned positively under stress (0/+), signaling stress‐triggered co‐regulation. Additionally, two pairs reversed from negative to positive relationships (−/+), potentially indicating synchronized responses vital for stress mitigation, such as detoxification or ion homeostasis.

A key case study involves GLYMA_09G185500 (encoding dehydrin) and GLYMA_15G021400 (encoding SIK2, a stress‐responsive kinase). Under standard conditions, these genes displayed an inverse relationship, likely reflecting distinct roles in baseline physiology. However, under abiotic stress, their correlation reversed to a positive one, suggesting integrated roles in stress adaptation. This dynamic shift underscores the adaptability of gene networks and emphasizes these genes as pivotal targets for engineering stress resilience.

Table 2 provides a complete listing of the examined gene pairs, including probe identifiers, correlation coefficients, significance levels for both control and stress treatments, and the Z‐score differences that quantify the magnitude of the correlation change.

### 3.9 Validation Through Comparative Genomics and Performance Evaluation

**FIGURE 6:**
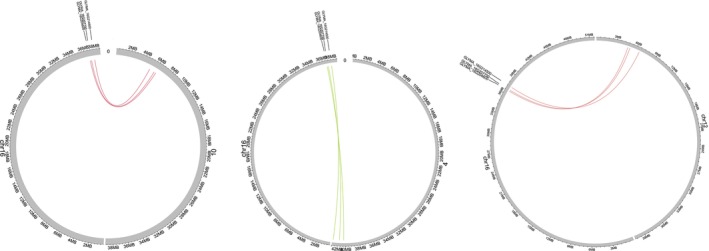
Comparative synteny maps of orthologous genes among four legume species ( *Glycine max* , *Phaseolus vulgaris* , *Vigna angularis* , and *Glycine soja* ).

Figure 6 reveals a highly preserved segment on soybean chromosome 16, housing three stress‐responsive gene loci: GLYMA_16G207700, GLYMA_16G204600, and GLYMA_16G214500. These genes demonstrate orthologous relationships with corresponding sequences in *Phaseolus vulgaris* and *Vigna angularis* , forming a syntenic haplotype cluster conserved across all four legume species. This shared genomic architecture implies evolutionary maintenance of stress‐adaptive mechanisms and structural stability in regions linked to abiotic stress tolerance, underscoring their utility in cross‐species genetic strategies for legume improvement.

The syntenic partners for these soybean genes include:

- In *Phaseolus vulgaris* : PHAVU_003G113200g, PHAVU_004G127500g, and PHAVU_004G139800g on chromosome 4.
- In *Vigna angularis* : LR48_Vigan10g057600, LR48_Vigan10g059800, and LR48_Vigan10g049500 on chromosome 10.

Functionally, the soybean gene trio plays critical roles in metabolic and stress‐related processes:

- GLYMA_16G207700 encodes 4,5‐DOPA dioxygenase extradiol (DODA), central to the synthesis of betalains and secondary metabolites. DODA‐related sequences may also mediate aromatic compound metabolism and oxidative stress mitigation.
- GLYMA_16G204600 produces enolase, a multifunctional enzyme involved in carbohydrate metabolism (e.g., glycolysis‐gluconeogenesis), amino acid production, and pathways tied to secondary metabolite synthesis.
- GLYMA_16G214500 is hypothesized to code for a disease resistance protein, which may aid in stress signal transduction and protective responses.

Ring‐cleaving dioxygenases, such as DODA, drive pivotal reactions in the aerobic breakdown of aromatic compounds. While many metabolic routes funnel into catecholic intermediates, others originate from noncatecholic hydroxy‐substituted aromatic acids (e.g., gentisate, salicylate, 1‐hydroxy‐2‐naphthoate, or aminohydroxybenzoates), underscoring the adaptability of DODA‐like enzymes in metabolic versatility and stress resilience.

Collectively, the retained synteny and functional interplay of these genes highlight their collaborative role in stress adaptation, positioning them as promising targets for comparative genomics, marker‐assisted selection, and inter‐species trait integration in legume crop development.

**FIGURE 7:**
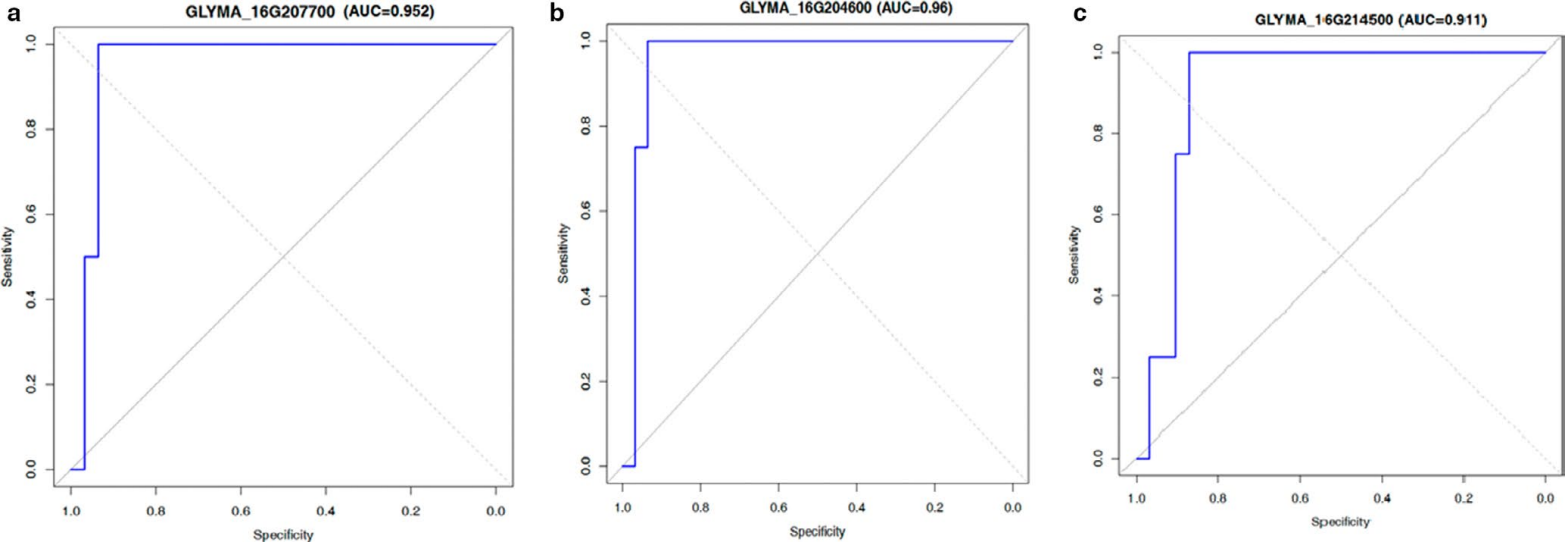
The ROC curves assess the performance of these three DEGs involved in abiotic stresses.

Our investigation uncovered a notable concentration of validated genes on chromosome 16 that synchronize their expression in response to environmental pressures. This genetic congregation suggests the existence of a chromosomal region that plays a pivotal role in orchestrating stress responses. Specifically, our analysis of genomic data revealed that a subset of genes on chromosome 16 is tightly linked and exhibits coordinated expression patterns when subjected to simulated drought conditions. This synergy implies a shared regulatory mechanism that enables these genes to work in tandem to enhance stress resilience. Notably, three of these genes demonstrated exceptional responsiveness to abiotic stress, displaying robust and consistent expression changes across diverse genetic backgrounds and tissue types. As a result, they emerge as prime candidates for conferring stress tolerance. Further validation using receiver operating characteristic (ROC) analysis confirmed the diagnostic potential of these genes, with GLYMA_16G207700, GLYMA_16G204600, and GLYMA_16G214500 exhibiting impressive area under the curve (AUC) values of 0.952, 0.96, and 0.911, respectively. The ROC curves for these three genes are illustrated in Figure 7, providing visual evidence of their potential as molecular biomarkers for abiotic stress.

**FIGURE 8:**
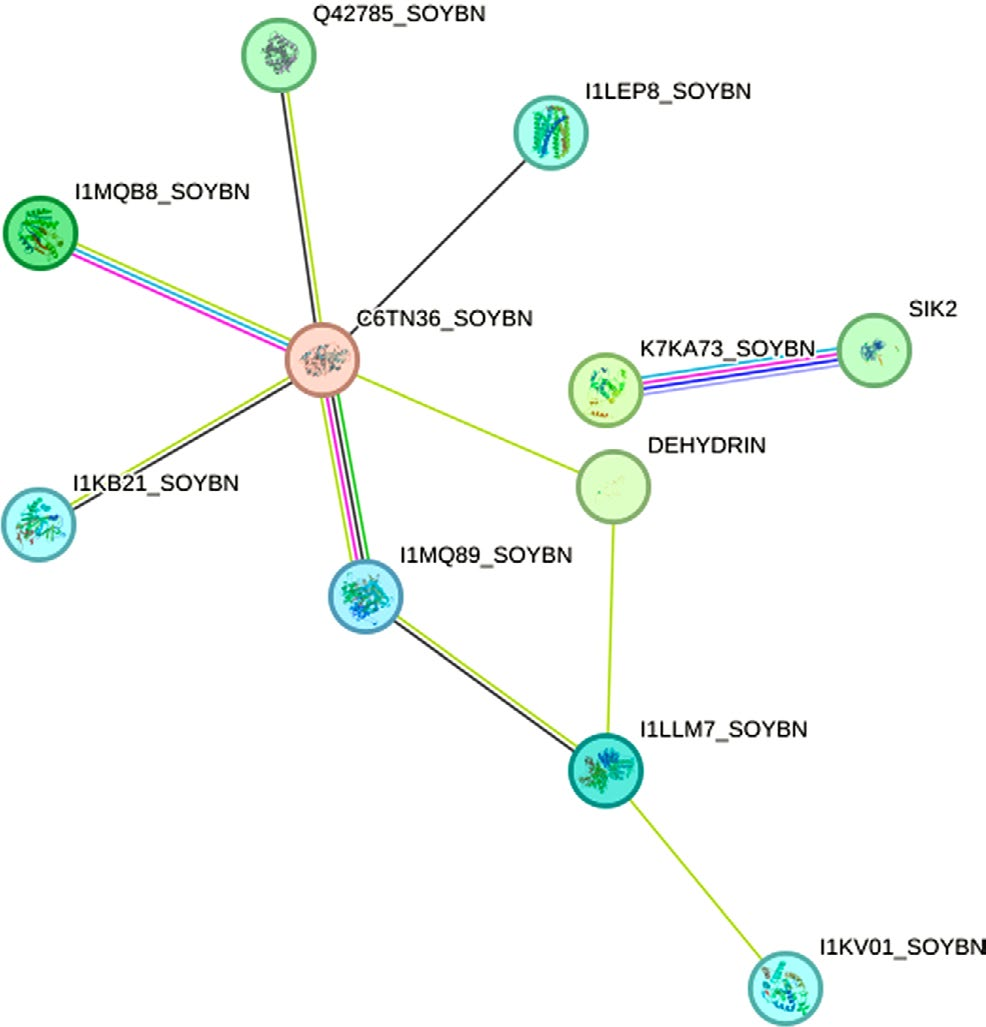
STRING‐derived protein–protein interaction (PPI) subnetwork of common genes identified under abiotic stress in soybean. The figure provides a simplified visualization of predicted functional associations among prioritized genes and is intended as an interaction overview rather than a formal hub‐gene detection or module–trait association analysis.

A complex web of interactions between proteins, known as the protein–protein interaction (PPI) network, was constructed using 37 genes that were consistently identified as differentially expressed across multiple analyses (as illustrated in Figure 8). This network was informed by a comprehensive database, STRING, which aggregates data from various sources, including experimental results, curated databases, and predictive models. Genes that emerged as common across different feature selection algorithms were designated as high‐priority candidates, owing to their strong predictive value and biological significance. These genes exhibited high connectivity within co‐expression and regulatory networks, suggesting that they serve as central hubs, orchestrating multiple downstream pathways and responding to diverse environmental stimuli. Moreover, their positions at the center of the network imply that they may play a role in maintaining its structure and functionality.

The common differentially expressed genes (DEGs) identified in this study are integral to various biological processes and stress responses, underscoring their importance in maintaining network integrity. Each of these genes contributes significantly to cellular resilience under stressful conditions. For instance, Glyceraldehyde‐3‐phosphate dehydrogenase, encoded by GLYMA_18G009700, is a key enzyme in the glycolytic pathway, which is essential for energy production and stress adaptation. Additionally, enolase, another enzyme involved in glycolysis, plays a crucial role in the biosynthesis of secondary metabolites and amino acids.

Other genes, such as asparagine synthetase, dehydrins, and salt‐inducible kinase 2, are also critical for plant adaptation to abiotic stressors, including drought, cold, and salt. Enolase, in particular, has been shown to play a role in plant immunity and development, in addition to its glycolytic function. Plant disease resistance proteins, including R proteins, are essential for detecting pathogens and triggering defense responses. These proteins recognize specific molecules produced by pathogens during infection and activate the plant's immune system. NBS‐LRR proteins, a class of intracellular R proteins, play a key role in recognizing pathogen effectors and initiating defense signaling pathways.

Furthermore, multiple plant detoxification genes, including plant UDP‐glycosyltransferases, have been identified as important for reducing toxicity and enhancing disease resistance. LigB domain‐containing protein, which serves as a building block for various plant compounds, including melanin and flavonoids, is also critical for plant stress responses. Nonsymbiotic hemoglobins, a class of plant proteins found in various tissues, are believed to play a role in responding to abiotic stress, as illustrated in Figure 8. Overall, the identification of these common DEGs has provided valuable insights into the complex networks that govern plant stress responses and has highlighted the importance of these genes in maintaining cellular resilience under stressful conditions.

## 4 Discussion

Traditional transcriptomic research into soybean abiotic stress often centers on identifying genes through simple differential expression (DEGs). In contrast, this study utilizes a multifaceted, network‐based framework that integrates DEG analysis with Differential Gene Correlation Analysis (DGCA), protein–protein interaction (PPI) data from STRING, and multi‐model machine learning. By moving past fold‐change metrics, our approach accounts for nuanced indicators like subtle shifts in gene expression, altered regulatory coordination, established functional biological context, and the stability of machine learning rankings. This robust methodology yields a high‐confidence shortlist for experimental validation. While our results partially align with established literature, we observe some divergence; this is an expected outcome of the inherent variability across transcriptomic studies. Factors such as differences in experimental design, sequencing depth, cultivar genetics, and normalization techniques necessarily influence gene identification. Furthermore, because our inclusion of DGCA highlights genes that undergo significant regulatory rewiring rather than just massive changes in expression levels, our findings naturally complement—and sometimes differ from—studies that rely exclusively on fold‐change thresholds (Khan 2025).

In this research, we utilized Differential Gene Correlation Analysis (DGCA) as a central method to pinpoint genes that displayed significant changes in their co‐expression networks when transitioning from control to stress environments. By mapping these shifts in transcript coordination, DGCA offered insights that moved beyond traditional differential gene expression (DGE) benchmarks. This approach is especially suited for studying abiotic stress, where the plant response is characterized by the synchronous regulation of various biological pathways rather than the simple activation of solitary genes. It is important to note that these co‐expression changes serve as statistical indicators of transcriptional reorganization rather than direct proof of underlying regulatory mechanisms. Consequently, DGCA acted as a filtering mechanism to prioritize genes linked to stress‐induced coordination.

To enhance the biological relevance of these findings, we integrated protein–protein interaction (PPI) data from the STRING database. While STRING does not account for condition‐specific dynamics, it serves as a robust foundation of prior knowledge, allowing us to assess the functional credibility of our candidate genes. By layering PPI context onto our transcriptional data, we successfully moved beyond criteria based solely on expression levels, focusing instead on genes sustained by both transcriptomic evidence and established protein‐level functional associations.

Furthermore, we refined our gene prioritization through a machine learning pipeline that employed both Random Forest and XGBoost algorithms, validated via cross‐validation. This ensemble strategy was designed to mitigate biases inherent to specific model architectures and to identify genes that maintained high importance scores across diverse computational settings. As with the correlation analysis, this machine learning ranking was used as a strategy for feature prioritization rather than a claim of mechanistic causality. Ultimately, the most promising candidates for abiotic stress responsiveness were those consistently identified by the machine learning models, further corroborated by the DGCA findings and their presence within functional PPI neighborhoods.

By synthesizing transcriptomic data across various soybean ( *Glycine max* ) cultivars, tissue types, and environmental pressures, this study identifies candidate genes that signify broad‐spectrum stress responses rather than isolated genotype‐specific reactions. The analytical pipeline incorporated a wide array of stressors—including osmotic agents (PEG and sugar), ionic imbalances (aluminum and salt), and nutrient fluctuations (cadmium, nitrogen, and phosphorus)—alongside control groups (Zhang 2022). This comprehensive approach facilitates a holistic understanding of how plants orchestrate transcriptomic shifts to survive diverse, yet often overlapping, environmental threats. However, such data diversity also brings inherent analytical challenges, necessitating a measured approach when interpreting differential expression patterns and correlation results.

From this multi‐layered framework, 37 core differentially expressed genes (DEGs) were isolated. Statistical validation via Fisher's exact test confirmed a non‐random clustering of these candidates on chromosomes 8 and 16. While this genomic concentration is significant, it does not confirm functional association, highlighting the need for further empirical investigation. Notably, three genes on chromosome 16—GLYMA_16G207700, GLYMA_16G204600, and GLYMA_16G214500—demonstrate functional consistency with orthologs found in *Vigna angularis* , *Phaseolus vulgaris* , and *Glycine soja* . Given that these genes coincide with previously identified quantitative trait loci (QTLs) and stress‐linked genomic intervals, they serve as promising targets for cross‐legume comparative studies and the development of molecular markers. Such findings suggest an evolutionary conservation of stress‐response mechanisms within the legume family, offering a roadmap for translational research.

Functional enrichment analysis of these candidates points to critical, biologically sound stress‐response pathways. For example, enolase—a key enzyme in glycolysis and gluconeogenesis—is not only vital for primary metabolism but also plays a documented role in abiotic stress adaptation (Wang et al. 2020). Previous research has consistently noted enolase expression shifts under salt, heavy metal, and drought stress, particularly in environments marked by high reactive oxygen species (ROS) activity (Tian et al. 2025). This aligns with our data, reinforcing the idea that enolase helps sustain metabolic balance and ROS mitigation (Kim et al. 2020).

Furthermore, the study indicates the involvement of betalain‐related pathways, which are well‐regarded for their antioxidant properties (Nakatsuka and Suetsugu 2009; Strack et al. 2003). Even though betalains are most often associated with species like *Hylocereus*, their role in vacuolar accumulation and cellular protection is a known mechanism of environmental resilience. The presence of these antioxidant‐related functions in our dataset underscores the importance of redox‐regulated pathways in plant defense. While our investigation did not empirically measure physiological indicators such as ion leakage or ROS levels, the strong identification of genes linked to osmotic regulation and antioxidant activity provides a robust framework for subsequent validation experiments (Tenore Tenore Basile 2012). These findings are further corroborated by syntenic relationships observed between soybean and *V. angularis* , suggesting that the stress‐responsive signatures identified here are rooted in conserved evolutionary pathways.

Despite the identified merits, certain constraints warrant mention. Primarily, this research is strictly computational and lacks wet‐lab verification; thus, the designated genes should be viewed as potential candidates rather than definitively proven stress mediators. Furthermore, the use of the *Glycine max* reference genome might impose limitations, given that this paleopolyploid species exhibits significant structural variation and gene duplication across different cultivars, which could impact sequencing alignment, annotation accuracy, and subsequent analysis. Additionally, Differential Gene Correlation Analysis (DGCA) is susceptible to issues related to dataset diversity, sample size, and correlation fluctuations, while machine learning models can be skewed by redundant features or hidden dataset biases. Finally, protein–protein interaction (PPI) data obtained from STRING reflects broad functional associations rather than specific, condition‐dependent interaction changes and depends heavily on existing database thresholds. These caveats highlight the necessity for conservative interpretation and emphasize the need for subsequent empirical testing and broader genomic comparisons.

Future efforts should prioritize the validation of these top‐tier candidates using tools like RT‐qPCR during various stages of stress, alongside functional testing such as gene editing, transgenic overexpression, or other perturbation techniques. Such investigations are vital to bridging the gap between transcriptomic and correlation‐based signals and actual physiological performance, ultimately revealing whether these genes truly bolster soybean stress resilience. Moreover, comparative studies involving other legumes could offer insights into the practical application of conserved genomic regions (Singh and Kumar 2019; Vitorino 2024). In this context, existing evidence of synteny—such as the NBS–LRR loci noted by Singh and Kumar and the agronomic loci discussed by Blair et al. 2012—underscores the potential of comparative genomics in advancing crop improvement.

Integrating differential expression profiling, network‐based correlation analysis, and machine learning prioritization has become a standard methodology for honing candidate gene selection in plant stress research. By synthesizing DEG analysis, DGCA, PPI mapping, and cross‐validated machine learning, this study presents a systematic computational workflow for identifying abiotic stress‐responsive genes in soybeans. This approach does not supplant traditional DEG studies but rather enhances them by layering transcript coordination and functional context onto the selection process. The resulting list of candidates provides a well‐founded set of hypotheses for future investigation, offering a valuable data‐driven foundation for breeding programs aimed at developing stress‐tolerant soybean germplasm once the results are empirically confirmed.

## 5 Conclusion

Enhancing soybean resilience to environmental stressors requires a deep dive into the underlying molecular mechanisms. This research utilized a network‐based computational approach to synthesize complex transcriptomic data, ultimately identifying 37 high‐priority genes linked to abiotic stress responses. By employing a hybrid methodology—combining Random Forest and XGBoost algorithms with Differential Gene Correlation Analysis (DGCA)—the study captured both predictive gene rankings and dynamic shifts in co‐expression patterns. This resulted in a refined gene list heavily involved in critical pathways such as amino acid production, energy regulation, and reactive oxygen species (ROS) management. Functional enrichment tests, including GO and KEGG, corroborated these findings, highlighting the roles of these genes in metabolic shifts like glycolysis and secondary metabolite synthesis—processes fundamental to how plants adapt to harsh conditions. Furthermore, the genetic signatures identified here often align with known stress‐associated chromosomal loci. The discovery of orthologous and syntenic relationships between these soybean genes and counterparts in *G. soja* , *P. vulgaris* , and *V. angularis* indicates that these stress‐response mechanisms are likely conserved across various legume species. However, as these findings are derived from computational modeling, they currently function as testable hypotheses rather than proven molecular drivers. To transition these results into practical agricultural applications, further experimental verification is essential. Upcoming research should focus on validating these candidates through RT‐qPCR, functional gene knockdown/overexpression studies, physiological testing, and haplotype–phenotype association mapping across broad soybean germplasm. Once confirmed, these biomarkers could become powerful tools for marker‐assisted breeding. Ultimately, this study establishes a robust, systematic framework for gene prioritization that paves the way for the development of more resilient soybean varieties.

## Funding

The authors have nothing to report.

## Ethics Statement

The authors have nothing to report.

## Consent

The authors have nothing to report.

## Conflicts of Interest

The authors declare no conflicts of interest.

## Supporting information


**Data S1:** It contains the full R codebase utilized to execute the integrated transcriptomic pipeline designed to pinpoint genes linked to abiotic stress in soybeans. This script encompasses essential steps, starting from raw data ingestion and preprocessing to comprehensive quality assurance and filtering of RNA‐seq expression data based on variance. To isolate genes with significant stress‐related responses, the analysis utilizes the *limma* package. Furthermore, the *DGCA* package is employed to track alterations in gene co‐expression networks that are specific to certain experimental conditions. To refine the selection of high‐priority candidate genes, the workflow incorporates machine learning‐driven feature selection via *Random Forest* and *XGBoost* algorithms, utilizing the *caret*, *randomForest*, and *xgboost* R packages. These models are optimized using five‐fold cross‐validation, with the area under the ROC curve serving as the primary performance benchmark. The eventual catalog of target genes is derived by synthesizing outputs from the differential expression analysis, co‐expression network shifts, and the most influential features identified by the machine learning models. Additionally, the script is configured to export summary reports detailing the gene counts at every processing stage, alongside the definitive list of key genes involved in the soybean abiotic stress response.

## Data Availability

Data available in article Data S1.
